# Rituximab for the treatment of refractory anti-glomerular basement membrane disease

**DOI:** 10.1080/0886022X.2022.2097405

**Published:** 2022-07-12

**Authors:** Xue-Fen Yang, Xiao-Yu Jia, Xiao-Juan Yu, Zhao Cui, Ming-Hui Zhao

**Affiliations:** a Renal Division, Peking University First Hospital, Beijing, China; b Renal Division, Shanxi Medical University Second Hospital, Shanxi Kidney Disease Institute, Taiyuan, China; c Institute of Nephrology, Peking University, Beijing, China; d Key Laboratory of Renal Disease, Ministry of Health of China, Beijing, China; e Key Laboratory of CKD Prevention and Treatment, Ministry of Education of China, Beijing, China; f Research Units of Diagnosis and Treatment of Immune-mediated Kidney Diseases, Chinese Academy of Medical Sciences, Beijing, China; g Peking-Tsinghua Center for Life Sciences, Beijing, China

**Keywords:** Rituximab, anti-glomerular basement membrane disease, severe, refractory

## Abstract

**Background:**

Anti-glomerular basement membrane (anti-GBM) disease is a rare but severe autoantibody-mediated immune disorder. The typical clinical presentation includes rapidly progressive glomerulonephritis and often concurrent pulmonary hemorrhage. The present study is aimed to investigate the therapeutic effects of rituximab either used alone or with other immunosuppressants.

**Methods:**

Eight patients diagnosed with anti-GBM disease and treated with rituximab from 2014 to 2020 were retrospectively reviewed.

**Results:**

Eight patients included 5 males and 3 females with a median age of 58.5 years. They all presented severe kidney injuries and 1 patient had lung hemorrhage. At diagnosis, the median of serum creatinine was 246 µmol/L (ranging from 91 to 850 µmol/L), with 3 patients requiring dialysis. All of them received corticosteroids and plasmapheresis. Rituximab was given as either standard four weekly doses or one pulse ranging from 100 to 600 mg. After a median follow-up of 34.5 months, kidney function was partially recovered or stabilized in 5/8 (62.5%) patients, free of dialysis. Anti-GBM antibodies remained undetected in all patients during follow-up. No severe adverse effect associated with rituximab was observed.

**Conclusion:**

Rituximab may be an alternative therapy in the treatment of patient with severe or refractory anti-GBM disease.

## Introduction

Anti-glomerular basement membrane (GBM) disease is a rare autoimmune disorder mediated by circulating autoantibodies that recognize cryptic epitopes within α345(IV) collagen in glomerular and alveolar basement membranes [1]. It has a strong genetic linkage to HLA-DRB1*1501 [2–4]. How the immune tolerance is disrupted and epitopes are exposed remains unknown. Some environmental factors have been found to be associated with the development of anti-GBM disease, such as infections, exposure to organic solvents or cigarette smoke, and lithotripsy treatment [1,5]. The typical clinical presentation includes rapidly progressive glomerulonephritis, and concurrent pulmonary hemorrhage.

Despite the rare occurrence of 0.5 ∼ 1.0 person per million of the general population per year [6], anti-GBM disease is the most aggressive form of glomerulonephritis. The predictors of poor prognosis include advanced age, oliguria, serum anti-GBM antibodies level, serum creatinine levels and chronic changes in renal biopsy [7]. Before the advent of plasmapheresis therapy, the mortality rate was over 90% [1]. The standard therapy of anti-GBM disease consists of the combination of corticosteroids, cyclophosphamide and plasmapheresis, which dramatically improved outcomes, especially when initiated at early stage [8]. However, despite standard therapy, less than one third of the patients survived with a preserved kidney function after 6 months follow-up [1]. Besides, the use of cyclophosphamide is associated with significant side effects including marrow suppression, risk of infection and hemorrhagic cystitis, secondary amenorrhea and high prevalence of cancer. Patientswho are refractory or intolerant to the standard therapy still have a poor prognosis of patient survival and kidney survival.

Anti-GBM disease is considered to be an autoantibody mediated disease [9]. Rituximab is a monoclonal chimeric antibody that targets the B lymphocyte antigen CD20. Depletion of B cells by rituximab could decrease antibody and cytokine production, and alter the process of antigen presentation [10]. Nevertheless, only a few cases have been reported describing the use of rituximab in anti-GBM disease. Due to the rarity and the fulminant course of anti-GBM disease, large randomized controlled trials to investigate therapeutic benefits of rituximab are difficult to conduct.

In this report, we described 8 cases of severe and/or refractory anti-GBM disease. They all received rituximab plus corticosteroids and plasmapheresis as an induction or maintenance therapy. No recurrences of antibodies or disease was observed after the treatment of rituximab during a median follow-up.

## Materials and methods

### Patients

A sequential of 8 patients with anti-GBM disease treated with rituximab were identified at Peking University First Hospital from January 2014 to February 2020. The diagnosis of anti-GBM disease was based on the presence of circulating anti-GBM antibodies examined by commercial ELISA kits (Euroimmun, Luebeck, Germany) and/or typical linear deposition of IgG along GBM in kidney biopsy excluding other causes including diabetes mellitus or paraproteinemia. ANCA were screened by indirect immunofluorescence assay (Euroimmun, Luebeck, Germany) and antigen-specific ELISA for anti-MPO antibodies and anti-PR3 antibodies in all patients (Euroimmun, Luebeck, Germany). The absolute count of peripheral B cells was measured using flow cytometry using anti-CD 20 antibody.

Clinical and pathological data were collected from medical records at the time of diagnosis and during follow-up. Severe disease was defined as dialysis dependence at diagnosis and/or the percentage of crescents >90% in glomerulus, or the need of mechanic ventilation assistance due to lung hemorrhage. Refractory disease was defined as failed response to standard therapy including antibody persistence or relapses, and/or complications to immuno-suppressive treatment. This study was conducted in accordance with the ethical principles stated in the Declaration of Helsinki and approved by the ethic committee of the Peking University First Hospital (number 2022 research 290-002).

### Treatment

Plasmapheresis (2–4 L) was performed daily or every other day until circulating anti-GBM antibodies were undetectable. All patients received intravenous methylprednisolone pulse therapy for 3 days, followed by prednisone (1 mg/kg per day) with gradual tapering. Cyclophosphamide was given 2 mg/kg per day orally or intravenously (usually 0.2 ∼ 0.4g/pulse per week). For frail or elder patients at higher risks of infections, methylprednisolone or cyclophosphamide were given at reduced dosages.

Rituximab was administered mainly by two regimens: (1) ‘Standard induction therapy’: rituximab was given at four weekly pulses at 375 mg/m^2^ either as the first choice of immunosuppressant, or an alternative one switching from cyclophosphamide. (2) ‘Reinforcement therapy’: one pulse of rituximab at various dosages (ranging from 100 to 600 mg) was given in addition to cyclophosphamide to reinforce the induction of refractory diseases.

### Kidney pathology

Kidney biopsy was performed in 6 patients at the time of diagnosis. Immunofluorescence was performed on frozen kidney sections using fluorescein conjugated rabbit/mouse anti-human IgG, IgA, IgM, C3c, C1q, FRA, albumin, light chain, IgG subclasses antibodies (Dako, Santa Clara, CA), and were evaluated under a fluorescence microscope (Nikon, Tokyo, Japan). Immunofluorescence staining intensity was graded as ‒ (negative), ± (weak), + (mild), ++ (moderate) and +++ (severe). For light microscopy, paraffin sections were stained with silver, periodic acid–Schiff, hematoxylin and eosin, and trichrome. For electron microscopy, biopsy materials were fixed in glutaraldehyde, post-fixed in osmium tetroxide, and embedded in Epon 812. Sections were stained with uranyl acetate and lead citrate. All the pathological data were reviewed independently by two kidney pathologists.

### Statistical analysis

SPSS statistical software (version 22.0, IBM) was used to perform the statistical analysis. Quantitative data were presented as mean ± SD or as median (range) as appropriate for continuous variables. Qualitative data were presented as number (%).

## Results

### Demographic and clinical features at presence

The demographic and clinical data of the 8 patients with anti-GBM disease were shown in Tables 1 and 2. Of the 8 patients, 5 were males and 3 were females, with a median age of 58.5 years. All except one patient (Patient 2) displayed prodromal infections before disease onset.

**Table 1.** The clinical data of the 8 patients with anti-GBM disease treated with rituximab.

Characteristic	Total patients (*N* = 8)
Gender (male/female)	5/3
Age (years)	58.5 (16–82)
Prodromal infection	7 (87.5%)
Hypertension	3 (37.5%)
Diabetes mellitus	0 (0%)
Oliguria/anuria	2 (25%)
Hemoptysis	1 (12.5%)
Gross Hematuria	3 (37.5%)
Proteinuria	7 (87.5%)
Nephrotic syndrome	2 (25%)
Albumin (g/L)	30.5 (22.1–34.8)
Kidney insufficiency	7 (87.5%)
SCr on diagnosis (µmol/L)	246 (91–850)
Anemia	6 (75%)
Hemoglobin (g/L)	102 (82–134)
ANCA	2 (25%)
MPO-ANCA/PR3-ANCA/both	2/0/0
Anti GBM antibodies (ELISA)	8 (100%)
Anti-GBM antibodies (U/mL)	46–>200
Treatment	
Corticosteroids	8 (100%)
Cyclophosphamide	5 (62.5%)
Plasmapheresis	8 (100%)
times	15 (3–17)
Rituximab	8 (100%)
Outcome	
Follow-up duration (months)	34.5 (15.5–84)
ESKD	3 (37.5%)
Death	0

Unless otherwise indicated, values are given as mean ± standard deviation, number (percentage), or median (range).

SCr: serum creatinine; ANCA: Anti-neutrophil cytoplasmic antibodies; MPO: Myeloperoxidase; PR3: proteinase 3; GBM: glomerular basement membrane; ESKD: end stage kidney disease.

**Tale 2.** Patients characteristics and treatment at the initiation of rituximab and at last follow-up.

	Patient 1 (F/62)	Patient 2 (F/63)	Patient 3 (M/75)	Patient 4 (F/55)	Patient 5 (M/37)	Patient 6 (M/16)	Patient 7 (M/82)	Patient 8 (M/54)
**Initiation**								
Creatinine (μmol/L)	144.5	83.7	649.4	305.3	361.5	229.6	706.9	555.6
Anti-GBM antibodies at diagnosis (U/mL)	>200	>200	46	>200	>200	189.3	>200	>200
Comorbidities	Pneumonia	RA, renal angiomyolipoma	chronic bronchitis, pneumonia	Pneumonia, pancytopenia	None	Leukopenia, thrombocytopenia	Pulmonary embolism, pneumonia	ILD, pneumonia, RF
**Kidney Biopsy**	Yes	Yes	Yes	Yes	Yes	Yes	No	No
crescents in glomerulus (%)	45.6	32	95.8	92.7	90.6	80	NA	NA
cellular crescents (%)	38.5	0	21.7	100.0	65.5	25	NA	NA
cellular-fibrous crescents (%)	57.7	50	69.6	0	34.5	75	NA	NA
fibrous crescents (%)	3.8	50	8.7	0	0	0	NA	NA
IgG linear deposition	++	++∼+++	++∼+++	+∼++	++∼+++	+++	NA	NA
C3 deposition	±	±	+	+++	++	+++	NA	NA
**Treatment**	MP 500 m*g* × 3 + Prednisolone 40 mg/d; CYC 3.1 g	MP 500 m*g* × 1 + 300 m*g* × 1 + 40 mg/d; CYC 9 g, then replaced by LEF 10 mg/d, and then AZA 100 mg/d	MP 500 m*g* × 2 + 200 m*g* × 2 + 40 mg/d + Prednisone 50 mg/d	MP 500 m*g* × 2 + 250 m*g* × 1 + Prednisolone 45 mg/d	MP 500 m*g* × 6 + Prednisone60 mg/d; CYC 50 mg/d	MP 500 m*g* × 3 + Prednisone 50 mg/d; CYC 8 g	MP 160 m*g* × 3 + 32 mg/d	MP 500 m*g* × 2 + 300 m*g* × 1 + Prednisone 20 mg/d; CYC 0.4 g
Number of Plasmapheresis sessions	7	16	3	15	16	15	17	9
Replacement fluids used for Plasmapheresis	Fresh frozen plasma and albumin	Fresh frozen plasma	Fresh frozen plasma and albumin	Fresh frozen plasma and albumin	Fresh frozen plasma and albumin	Fresh frozen plasma and albumin	Fresh frozen plasma and albumin	Fresh frozen plasma
Dialysis at diagnosis	No	No	Yes	No	No	No	Yes	Yes
Time from diagnosis to RTX treatment	4.2 months	7 months	1 month	1 month	1.6 months	10 months	2 months	26 months
Time from last Plasmapheresis to RTX treatment	3.6 months	5.8 months	0.7 month	1 day	4 days	9 months	7 days	25 months
Indication of rituximab	Refractory disease	Refractory disease	Severe disease	Severe disease	Severe disease	Refractory and severe disease	Severe disease	Refractory and severe disease
Rituximab dosage	375 mg/m^2^ ×5	375 mg/m^2^ ×4	375 mg/m^2^ ×4	375 mg/m^2^ ×7	300 mg ×1	600 mg ×1	100 mg ×1	100 mg ×1
B cell counts after RTX treatment (cells/μL)	0	0	0	0	1.74	NA*	0	0.44
**Outcome**								
Follow-up duration (months)	15.5	84	42.5	27	33	57.5	36	29
anti-GBM antibodies (U/mL)	negative	negative	negative	negative	negative	negative	NA*	negative
Serum creatinine at the end of follow-up	Creatinine 123.4 μmol/L	Creatinine 109 μmol/L	Dialysis dependent	Creatinine 134 μmol/L	Creatinine 300 ∼ 400 μmol/L	Creatinine 133.5 μmol/L	Dialysis dependent	Dialysis dependent

M: male; F: female; GBM: glomerular basement membrane antibody; ILD: Interstitial lung disease; RF: respiratory failure; RA: rheumatoid arthritis; IgG: immunoglobulin G; MP: methylprednisolone; CYC: cyclophosphamide; LEF: leflunomide; AZA: azathioprine; RTX: rituximab; NA: not applicable; NA*: data not available.

One patient (Patient 3) presented concurrent pulmonary hemorrhage. The median serum creatinine on diagnosis was 246 µmol/L (range 91–850 µmol/L). 7/8 (87.5%) patients presented kidney insufficiency (serum creatinine > 133 µmol/L), and 3 (Patients 3, 7 and 8) of them (3/8, 37.5%) were dialysis dependent at diagnosis. Oliguria/anuria was present in 2/8 (25%) patients. Gross hematuria was observed in 3/8 (37.5%) patients, and proteinuria in 7/8 (87.5%) patients with 2 of them presenting nephrotic syndrome. All patients were positive for circulating anti-GBM antibodies (range 46 to >200, normal <20 U/mL) detected by ELISA. Two patients also possessed positive ANCA (Patients 3 and 8), both recognizing MPO.

Three patients (Patients 3, 7 and 8) had concomitant pneumonia at presence. Two patients (Patients 7 and 8) were not eligible for performing a kidney biopsy because of the severe condition. Patient 8 had concomitant grand mal epilepsy, nephrorrhagia, intracerebral hemorrhage and splenorrhagia. Patient 7 had concomitant pulmonary embolism and received anti-coagulation therapy.

### Kidney pathology

Six of the 8 patients underwent kidney biopsy at diagnosis (Table 2), and typical linear deposits of IgG along GBM was demonstrated in all of them. All of the patients showed crescent formation with a median percentage of crescents in glomeruli of 85.3% (32 to 95.8%). Four of them (Patients 3, 4, 5 and 6) had diffuse crescents occupying >50% of the glomeruli. Three patients showed IgA deposition in the mesangium area by Immunofluorescence and electron dense deposits in the corresponding area under electron microscopy. All six patients showed tubular atrophy and interstitial fibrosis, interstitial inflammatory cells infiltration. One patient (Patient 1) showed arteriolosclerosis.

### Treatment and clinical response

All patients received methylprednisolone pulse therapy, followed by methylprednisolone or prednisone (0.8 ∼ 1 mg/kg per day), and then gradual tapering. Of notice, 5 patients received reduced pulses of methylprednisolone (160 ∼ 300 mg per day) due to co-infections and/or leukopenia.

Four patients (Patients 1, 2, 3 and 4) received four weekly pulses of rituximab as induction therapy at 375 mg/m^2^, because of severe/refractory disease or comorbidities. Patient 1 switched from cyclophosphamide to rituximab for induction therapy due to pneumonia, when cyclophosphamide at a cumulative dose of 3.1 g. Patients 2 showed persistent positive anti-GBM antibodies despite a rigorous induction therapy, including pulses of methylprednisolone, 16 sessions of plasmapheresis, and an accumulative dose of cyclophosphamide at 9 g followed by leflunomide and azathioprine. At 7 months after diagnosis, she received four weekly rituximab, and gradually discontinued all immunosuppressant drugs when antibody became negative. Patient 3 was a 75 years old male with positive ANCA, who presented severe disease with 95.8% of crescent formation on kidney biopsy and pulmonary hemorrhage, as well as pneumonia at diagnosis. He received rituximab plus plasmapheresis and steroids as the initial induction therapy. Patients 4 had 92.7% of crescents on kidney biopsy, rapidly deteriorated kidney function and persistent positive anti-GBM antibodies after pulses of methylprednisolone and 15 sessions of plasmapheresis. She also suffered from pancytopenia and pneumonia, and thus received rituximab as the initial immunosuppressant.

The remaining 4 patients (Patient 5, 6, 7 and 8) received one pulse of rituximab as ‘Reinforcement therapy’ due to refractory diseases. Patient 5 had 90.6% of crescents on kidney biopsy. He received two rounds of methylprednisolone pulse therapy (500 mg/day for 3 days for each time), 15 sessions of plasmapheresis and oral cyclophosphamide (50 mg/day) before anti-GBM antibodies finally became negative. However, serum creatinine still increased from baseline, and one pulse of rituximab at 300 mg was then administered. Patient 6 also received the standard therapy (steroids, cyclophosphamide and PE), but showed a relapse with recurrent anti-GBM antibodies when prednisone was tapered to 10 mg/d and the total amount of cyclophosphamide at 8 g. He then received one pulse of rituximab at 600 mg. Patient 7 was a senile patient (>80 years old), who presented with a high level of anti-GBM antibodies and advanced kidney disease with leukopenia and pneumonia. Given the risk of infections and poor kidney prognosis without lung involvement, he received one pulse of rituximab (100 mg) with steroids and plasmapheresis, but no cyclophosphamide. Patient 8 had concurrent ANCA, and treated with a standard induction therapy of steroids, plasmapheresis and cyclophosphamide. The patient progressed to ESKD and went on maintenance hemodialysis. At 22 months after diagnosis, he experienced a rare and severe relapse of disease with positive anti-GBM antibodies (66 U/ml) but undetectable ANCA, and suffered from severe organ bleeding including intracerebral hemorrhage resulting in grand mal and splenorrhagia, pneumonia and respiratory failure requiring mechanic ventilation assistance at the same time. He received one pulse of rituximab (100 mg), in addition to steroids and cyclophosphamide (Table 2).

### Outcome

All of the 8 patients in the present study had severe and/or refractory diseases. The follow-up time ranged from 15.5 to 84 months with a median of 34.5 months. No recurrences of antibodies or disease was observed after the treatment of rituximab during follow-up.

Three patients (patients 3, 7 and 8), who required kidney replacement therapy at the initiation of rituximab, progressed to ESKD. Three patients (patients 1, 4 and 6) had a partial recovery of kidney function, and 2 patients (patients 2 and 5) remained stable and free of dialysis. No deaths or serious infections occurred in any of the 8 patients. Patient and kidney survival were 100% and 62.5% respectively.

## Discussion

In the present study, we reported the outcomes of 8 severe or refractory anti-GBM disease treated with rituximab, two of whom received rituximab as the initial immunosuppressant alone. After a mean follow-up of 34.5 months, kidney function was partially recovered or stabilized in 5/8 (62.5%) patients, free of dialysis. No severe adverse reactions were observed.

Rituximab has been mentioned in the 2021 KDIGO Clinical Guidelines for the treatment of anti-GBM disease [11]. However, due to the limited number of cases, no recommendation level was given. Case reports or small, uncontrolled series of anti-GBM disease treated with rituximab have been published previously [12–18]. Touzot et al. [19] reported the outcomes of eight anti-GBM patients treated with rituximab. Seven of the 8 patients achieved complete remission. Patient and kidney survival was 100% and 75% respectively after a mean follow-up of 25.6 months, though eGFR did not improve. Severe bacterial infection occurred in 1/8 patient. However, all the enrolled 8 patients of Touzot et al. received intravenously cyclophosphamide as the first line treatment. Rituximab was associated with cyclophosphamide for 3 patients and mycophenolate mofetil for 2 at the same time. In the present study, we reported a similar result in patient and kidney survival (100% and 62.5%) of anti-GBM patients treated with rituximab, and no adverse effects. Due to the rare incidence, it is difficult to conduct randomized controlled trials to investigate the therapeutic effects of rituximab verse cyclophosphamide. Rituximab as an initial immunosuppressant without cyclophosphamide had only been described in individual cases [9,20–22]. Three patients of our series received rituximab as the initial immunosuppressant for induction therapy, receiving no cyclophosphamide (Patient 3 and 4) or a prior small amount of dosage (Patient 1). Collectively, our study and the previous cases suggested that rituximab may be an alternative treatment option for the induction and maintenance therapy in anti-GBM disease, especially for patients with contraindications of cyclophosphamide and refractory or recurrent patients. Rituximab can be administered as four weekly pulses of 375 mg/m^2^ or a single pulse regimen.

In our cohort, rituximab did not prevent the evolution to ESRD for 3 patients, who required hemodialysis at the initiation of rituximab. Similar results have been reported in the previous studies [15,20,23]. However, some case reports showed that patients were able to temporarily withdraw from hemodialysis [9,21]. Rituximab exerted its therapeutic effects by targeting B cells to suppress the formation of new anti-GBM antibodies in anti-GBM disease [10]. Serum creatinine at the initiation is related to the long-term kidney survival. Therefore, it is rational to recommend early initiation of rituximab in patients with refractory anti-GBM disease.

There is no generally accepted standard schedule for rituximab therapy in anti-GBM disease. The regimen of rituximab received by anti-GBM patients in reported studies varied. A standard protocol of four weekly doses or B-cell driving therapy are both currently used for treating membranous nephropathy or vasculitis with rituximab. In our cohort, four patients received only one pulse of rituximab as reinforcement therapy due to refractory diseases, which accelerated the clearance of antibodies or the tapering of steroids and other immunosuppressants. Though kidney outcome did not improve significantly, tailored therapy of rituximab might be implemented in the treatment of severe or refractory anti-GBM disease. Future clinical trials or large series of observational studies are merited to provide further evidence.

In addition to the efficacy, rituximab also showed a favorable safety profile compared to other immunosuppressant used in the treatment of anti-GBM disease. In our series, four patients were older than 60 years, five patients had pneumonia, and two patients had leukopenia. All patients showed well tolerance of rituximab and no associated infections were found during follow up. In a retrospective study [24], the outcomes of 98 patients receiving rituximab for glomerular disease were reviewed in eight French nephrology departments. An infection rate they reported was 21.6 per 100 patient-years. The independent risk factors included cumulative rituximab dose, concomitant use of azathioprine and presence of diabetes mellitus. Besides, kidney failure was significantly associated with an increased infection risk. However, none of the 98 patients enrolled in the study were diagnosed with anti-GBM disease. Further studies are needed to clarify the risk-benefit of rituximab compared to conventional therapy in the treatment of anti-GBM disease.

In conclusion, our study suggested that rituximab may be a safe alternative therapeutic option for the induction and maintenance treatment of severe and refractory anti-GBM disease, especially in patients with contraindications to cyclophosphamide.

## Data Availability

The raw data supporting the conclusions of this article will be made available by the authors, without undue reservation.
